# Heart and Cardiac Substructure Dose Sparing in Synchronous Bilateral Breast Radiotherapy: A Dosimetric Study of Proton and Photon Radiation Therapy

**DOI:** 10.3389/fonc.2019.01456

**Published:** 2020-01-10

**Authors:** Tao Sun, Xiutong Lin, Ying Tong, Xiao Liu, Lingjing Pan, Cheng Tao, Jinghao Duan, Yong Yin

**Affiliations:** ^1^Department of Radiation Physics, Shandong Cancer Hospital and Institute, Shandong First Medical University and Shandong Academy of Medical Sciences, Jinan, China; ^2^Department of Radiation Oncology, Hefei Ion Medical Center, Hefei, China

**Keywords:** synchronous bilateral breast cancer, intensity-modulated radiation therapy, volumetric-modulated arc therapy, tomotherapy, proton beam therapy, dosimetry

## Abstract

**Background:** Synchronous bilateral breast cancer (SBBC) is rare. The purpose of this study was to compare the dosimetric differences in intensity-modulated radiation therapy (IMRT), volumetric-modulated arc therapy (VMAT), helical tomotherapy (HT), and intensity-modulated proton therapy (IMPT) to find an optimal radiotherapy technique for bilateral breast cancer radiotherapy.

**Methods:** For 11 patients who received synchronous bilateral whole-breast irradiation without local lymph nodal regions, six plans were designed for each patient: IMRT with a single isocenter (IMRT-ISO1), IMRT with two isocenters (IMRT-ISO2), VMAT with a single isocenter (VMAT-ISO1), VMAT with two isocenters (VMAT-ISO2), HT, and IMPT. The differences between the single- and dual-isocentric plans for IMRT and VMAT were compared, and the plan with the better quality was selected for further dosimetric comparisons with IMPT and HT. The plan aimed for a target coverage of at least 95% with the prescription dose of 50 Gy [relative biological effectiveness (RBE)] while minimizing the dose of organs at risk (OARs).

**Results:** IMRT-ISO1 and VMAT-ISO2 plans were adopted for further dosimetric comparisons because of the reduced dose of the heart and/or lungs compared to IMRT-ISO2 and VMAT-ISO1 plans. The dose coverage of the planning target volume (PTV) was significantly higher in IMPT plans than that in all other plans. VMAT and IMPT plans showed the best conformity, whereas IMRT plans showed the worst conformity. Compared to IMRT and VMAT plans, IMPT and HT plans achieved significantly higher dose homogeneity. IMPT plans reduced the mean dose and low dose volume (V_5_, V_10_, and V_20_) of the heart, left anterior descending artery (LAD), and left ventricle (LV). In high-dose volumes of the heart and cardiac substructures, the IMPT, VMAT, and HT techniques showed similar advantages, and IMRT plans increased the values more than other techniques. IMPT plans had the maximal lung and normal tissue sparing but increased the skin dose compared to IMRT and VMAT plans.

**Conclusions:** IMPT plans improve both the target coverage and the OARs sparing, especially for the heart, cardiac substructures (LAD and LV), lungs and normal tissue, in synchronous bilateral breast radiotherapy. VMAT and HT could be selected as suboptimal techniques for SBBC patients.

## Background

Breast cancer is the most common malignancy among women worldwide. The risk of local recurrence is halved, and the mortality of breast cancer is reduced by one-sixth in patients with breast cancer who receive adjuvant radiotherapy (1). However, radiotherapy is related to long-term cardiopulmonary toxicity and the risk of secondary malignancy, and cardiac toxicity may reduce survival (2–4).

Cardiovascular disease after radiation therapy has become the leading cause of non-breast cancer death in breast cancer patients (5). Therefore, the cardiotoxicity caused by radiotherapy is an important problem that needs to be studied extensively. Darby et al. (2) found that there was a linear relationship between the mean heart dose (MHD) and the incidence of ischemic heart disease, which increased by 7.4% per Gy of the MHD. Therefore, reducing the MHD is critical to reducing long-term cardiotoxicity. Most studies have found that the cardiotoxicity induced by radiotherapy is also closely related to the dose of key heart substructures, including the left anterior descending artery (LAD) and the left ventricle (LV), because studies have shown that high-grade coronary stenosis in LAD is increased in women receiving radiation for the left breast, suggesting a direct link between radiotherapy and coronary stenosis, so the dose to LAD is particularly important (6–9). Therefore, it is important to reduce the dose of the heart and cardiac substructures in patients receiving breast radiotherapy.

It is estimated that synchronous bilateral breast cancer (SBBC) accounts for 2.1% of all breast cancers (10). Compared to unilateral breast cancer, SBBC has a wider treatment volume, and it is closer to the lungs and heart. Because of these anatomical characteristics, designing a plan for radiotherapy for SBBC is a time-consuming and complex process. Three-dimensional conformal radiotherapy (3D-CRT) with tangential fields has some drawbacks for SBBC. Traditional tangential fields may overlap at the anterior border of the sternum, resulting in a high dose in the area in front of the sternum. The inhomogeneity of the target and poor cosmetic effects can be observed in 3D-CRT. Compared to 3D-CRT, intensity-modulated radiation therapy (IMRT), volumetric-modulated arc therapy (VMAT), or tomotherapy can improve the target dose coverage and can achieve acceptable cosmetic effects and cardiopulmonary sparing. Thus, the use of VMAT, IMRT, or tomotherapy was strongly suggested for SBBC (11–15). However, few studies have compared dosimetric differences between the three techniques of IMRT, VMAT, and tomotherapy in SBBC.

Past dosimetric studies have shown that proton therapy delivered a lower cardiac dose compared to photon techniques in unilateral breast cancer (16–19). Intensity-modulated proton therapy (IMPT) has the advantage of improving the dose distribution and reducing the dose of organs at risk (OARs) more without being limited by complex anatomical structures. However, the past studies did not include a dosimetric comparison forSBBC.

The aim of this study was to compare the dosimetric differences of IMRT, VMAT, tomotherapy and IMPT techniques and to establish optimal solutions with heart and substructure sparing for SBBC.

## Methods

### Patient Selection and Volume Delineation

From September 2006 to December 2018, patients who were diagnosed with SBBC and received bilateral breast radiotherapy at Shandong Cancer Hospital were identified. CT planning datasets of 11 patients (stage T1–T2; post-lumpectomy) were retrieved for dosimetric comparisons. This study was approved by the research ethics board of the Shandong Cancer Hospital.

The clinical target volume (CTV) for the bilateral breast was contoured according to the Radiation Therapy Oncology Group contouring atlas group (20). The planning target volumes (PTVs) were created by expanding a 5-mm margin to the CTV. All PTVs were clipped 5 mm from the skin surface. The whole lung, heart, LV, LAD, liver, and skin were considered organs at risk (OARs). The normal structures were defined as the body minus the PTV (B-P). The skin was defined as a 3-mm area under the body outside of the PTV.

### Treatment Planning

For each patient, six plans were generated: IMRT with a single isocenter (IMRT-ISO1), IMRT with two isocenters (IMRT-ISO2), VMAT with a single isocenter (VMAT-ISO1), VMAT with two isocenters (VMAT-ISO2), helical tomotherapy (HT), and IMPT.

IMRT and VMAT plans were designed with the Eclipse version 13.6 treatment planning system (Varian Medical Systems, Palo Alto, CA, USA) using a Varian Trilogy linear accelerator. Plans were optimized using a progressive resolution optimization (PRO) algorithm, and the dose calculation algorithm was an analytic anisotropic algorithm (AAA) with a 0.25-cm grid size. Six MV photon beams were used for the two plans.

For IMRT-ISO1 plans, a single isocenter was placed under the middle of the sternum. Two tangential photon beams were designed for each breast. If the chest wall was too curved, 1–2 additional segment fields were added to protect the lungs and heart. For IMRT-ISO2 plans, two isocenters were placed in the center of the half circle formed by the lung-breast interface at the same CT section. The field angles were the same as those in the IMRT-ISO1 plans.

The isocenters of the VMAT plans were the same as those of the IMRT plans. A bowtie technique, as described by Viren et al. (21), was employed. Four partial arcs, each consisting of ~80° of rotation, were used for one breast. A total of eight partial arcs were designed for the VMAT-ISO1 and VMAT-ISO2 plans. The medial x-jaw shielding the lungs and heart was set to the minimum site (−2 cm), and the lateral x-jaw was opened to include all breast tissue in the beam's-eye-view (BEV). The appropriate starting and stopping angles were selected according to the anatomy of individual patients. To minimize the exposure of the lungs and heart, paring apple-like tangential beams were designed for VMAT plans. VMAT-ISO1 and VMAT-ISO2 plans used different arc degrees to obtain the lowest OARs dose.

HT plans were designed in the Tomotherapy version 5.1.3 treatment planning system (Accuray® Planning Station, Madison, WI, USA). A 2.51-cm field width and a 0.287 pitch were used in the plan. The modulation factor was initially set to 3 and was adjusted throughout optimization. Most areas behind the bilateral breast, including most of the lungs and heart, were all directly blocked for planning.

The IMPT plans were generated in the Varian Eclipse ProBeam proton system. To maximize the robustness of IMPT plans, two en face fields (45/315°) were designed for the bilateral breast. The en face beams were in the direction of respiratory movement, thus reducing the risk of target coverage loss caused by respiratory movement. A range shifter with a thickness of 5 cm was used in the proton fields to cover the superficial target areas at the surface. In IMPT plans, multiple-field optimization and selective robust optimization (22) were used. CTV plus robustness optimization with a 5-mm setup uncertainty and 3.5% range uncertainty was used, whereas normal objectives were applied to the PTV. Plans were generated using a non-linear universal proton optimizer algorithm (NUPO) (23). A proton convolution superposition algorithm was used for the dose calculation with a 0.25-cm grid size.

The prescribed dose was 50 Gy in 2-Gy fractions of relative biological effectiveness (RBE). Plans were designed to first achieve at least 95% coverage of the target to a dose of 50 Gy (RBE) and second to achieve maximal sparing of the OARs, especially the lungs, heart, and substructures. Doses to the OARs were minimized without compromising the PTV coverage. Optimization was performed to reduce the mean total-lung dose (MLD) and mean heart dose (MHD), as well as the mean dose to the LAD. The volume of PTV wrapped in 110% of the prescription dose was <1%. The plan objectives were to implement V_20Gy_ ≤30% for both lungs (24) and an MHD ≤ 5 Gy (3) while synchronously reducing the dose to the OARs as much as possible.

### Dosimetric Evaluation

The dose statistics of the plans were based on dose-volume histogram (DVH) analysis. For PTV, D_2_, D_98_ (the dose of 2% and 98% volume of PTV) and the values of V_100_ and V_110_ (the volumes receiving 100% and 110% of the prescribed dose) were analyzed. The conformity index (CI) and homogeneity index (HI) of the PTV were analyzed. The CI was defined as follows:

$$\begin{array}{l} CI=\frac{{TV_{PV}}^{2}}{TV\times PV} \end{array}$$

The TV_PV_ represents the volume of the PTV covered by the prescribed dose, TV represents the volume of the PTV, and PV represents the total volume covered by the prescribed dose (25).

The HI was defined as follows:

$$\begin{array}{l} HI=\frac{D_{2}-D_{98}}{D_{p}} \end{array}$$

D_2_ represents the dose of 2% of the volume of the PTV, and D_98_ represents the dose of 98% of the volume of the PTV. D_p_ represents the prescribed dose (26). Lower HI values indicate more homogeneous target doses.

To evaluate the irradiated dose to the OARs, the analysis included the mean dose and V_XGy_ (OARs volume receiving X Gy). For the whole lung, V_5Gy_, V_10Gy_, V_20Gy_, V_30Gy_, V_40Gy_, and the mean dose were compared. The V_5Gy_, V_10Gy_, V_20Gy_, V_30Gy_, V_40Gy_, and D_mean_ for the heart and LV were compared. V_5Gy_, V_10Gy_, V_20Gy_, V_30Gy_, V_40Gy_, D_max_, and D_mean_ were also compared for the LAD. The V_5Gy_, V_10Gy_, V_20Gy_, V_30Gy_, V_40Gy_, V_50Gy_, and D_mean_ for B-P and V_30Gy_, V_40Gy_, V_45Gy_, V_50Gy_, and D_mean_ for the skin were analyzed. The D_mean_ for the liver was analyzed. The monitor units (MUs) for the IMRT and VMAT plans were compared.

First, the differences between the single- and dual-isocentric plans for IMRT and VMAT were compared, and the plan with the higher quality was selected from the IMRT and VMAT plans for further dosimetric comparisons with IMPT and HT.

### Statistical Analysis

All data were analyzed using the Statistical Package for Social Sciences v19.0 software (SPSS Inc., Chicago, IL, USA). The Wilcoxon matched-paired signed-rank test was used to evaluate the significance of the observed differences between the single- and dual-isocentric plans for IMRT and VMAT. One-way analysis of variance with Tukey's multiple comparison *post hoc* test was applied to compare the four techniques. The differences were considered statistically significant when *p* < 0.05.

## Results

### PTV

The average PTV was 1,364.1 ± 302.0 cm^3^, ranging from 966.8 to 1,916.6 cm^3^.

### Dose Comparisons for IMRT and VMAT Plans

Table 1 shows the dose parameters for the ISO1 and ISO2 plans of IMRT and VMAT. No significant differences were detected between the ISO1 and ISO2 plans for IMRT and VMAT in regard to the dose coverage, D_2_, and D_98_ of the PTV. The IMRT-ISO1 plans achieved significantly higher dose homogeneity (*p* < 0.05) than the IMRT-ISO2 plans. Significant heart dose (V_10_, V_20_, V_30_, V_40_, and D_mean_) sparing was achieved with IMRT-ISO1 plans compared to IMRT-ISO2 plans. No significant differences were detected in the dose of the lungs, LV, LAD, liver, and MUs between the IMRT-ISO1 and IMRT-ISO2 plans. Among the VMAT-ISO1 and VMAT-ISO2 plans, the VMAT-ISO2 plans showed higher conformity. Lung doses (V_10_, V_20_, V_30_, V_40_, and D_mean_), heart doses (V_20_, V_30_, and V_40_), and LV (D_mean_) and LAD (D_mean_ and D_max_) doses were significantly reduced in VMAT-ISO2 plans. No statistically significant differences were found in the V_5_ of lungs, V_10_ and D_mean_ of the heart, and D_mean_ of the liver and MUs between VMAT-ISO1 and VMAT-ISO2 plans. However, VMAT-ISO2 plans showed higher values on the V_5_ of the heart.

**Table 1:** Summary of dosimetric analysis for ISO1 and ISO2 plans of IMRT and VMAT.

	**IMRT-ISO1**	**IMRT-ISO2**	***p***	**VMAT-ISO1**	**VMAT-ISO2**	***p***
**PTV**						
V_100%_ (%)	96.9 ± 0.6	96.4 ± 0.9	0.138	97.0 ± 0.7	96.4 ± 0.8	0.139
D_2_ (cGy)	5,421.7 ± 32.3	5,427.1 ± 44.9	0.374	5,445.7 ± 22.2	5,435.9 ± 32.9	0.790
D_98_ (cGy)	4,918.7 ± 53.8	4,870.6 ± 94.0	0.091	4,903.8 ± 55.5	4,861.9 ± 78.3	0.155
CI	0.77 ± 0.04	0.77 ± 0.04	0.473	0.80 ± 0.05	0.84 ± 0.02	**0.006**
HI	0.10 ± 0.01	0.11 ± 0.02	**0.047**	0.11 ± 0.01	0.11 ± 0.02	0.230
**Lungs**						
V_5_ (%)	30.2 ± 4.2	30.8 ± 4.3	0.143	35.0 ± 3.7	33.9 ± 3.4	0.139
V_10_ (%)	21.0 ± 3.6	21.6 ± 4.0	0.062	23.5 ± 3.5	20.5 ± 3.6	**0.033**
V_20_ (%)	15.6 ± 3.5	16.1 ± 3.8	0.068	15.0 ± 3.4	10.2 ± 2.5	**0.003**
V_30_ (%)	11.4 ± 4.1	11.6 ± 4.3	0.358	10.3 ± 3.6	6.0 ± 2.5	**0.018**
V_40_ (%)	9.0 ± 3.3	8.8 ± 3.3	0.333	7.4 ± 3.0	2.8 ± 1.3	**0.003**
D_mean_ (cGy)	881.8 ± 147.0	888.6 ± 150.8	0.213	895.5 ± 136.1	719.1 ± 89.2	**0.003**
**Heart**						
V_5_ (%)	16.5 ± 10.4	16.8 ± 10.7	0.305	16.9 ± 5.3	29.5 ± 8.3	**0.004**
V_10_ (%)	9.2 ± 5.9	10.7 ± 7.7	**0.041**	10.1 ± 4.0	11.7 ± 6.1	0.286
V_20_ (%)	6.1 ± 3.6	6.8 ± 3.9	**0.028**	6.2 ± 3.2	2.4 ± 2.1	**0.004**
V_30_ (%)	3.9 ± 3.3	4.6 ± 3.6	**0.012**	3.8 ± 2.4	0.8 ± 1.3	**0.005**
V_40_ (%)	3.0 ± 2.6	3.4 ± 3.0	**0.038**	1.7 ± 1.5	0.1 ± 0.2	**0.005**
D_mean_ (cGy)	498.4 ± 184.7	526.2 ± 189.9	**0.041**	496.1 ± 134.7	484.7 ± 108.0	0.929
**LV**						
D_mean_ (cGy)	778.1 ± 276.9	818.6 ± 279.7	0.155	782.9 ± 250.4	455.8 ± 129.8	**0.003**
**LAD**						
D_mean_ (cGy)	2,149.7 ± 857	2,187.7 ± 899.1	0.790	2,130.1 ± 934.8	1,436.1 ± 576	**0.003**
D_max_ (cGy)	4,725.0 ± 504.6	4,636.7 ± 399.6	0.155	4,452.0 ± 416.8	3,886.8 ± 727.2	**0.004**
**Liver**						
D_mean_ (cGy)	400.1 ± 140.0	402.4 ± 138.6	0.722	404.6 ± 144.7	363.8 ± 99.5	0.213
**MU**	*1, 344*±157.4	1,362.6 ± 344.6	0.657	987.2 ± 162.2	1,113.2 ± 118.4	0.110

*PTV, planning target volume; V_x%_, volume receiving at least x% of the prescribed dose; D_x_, dose received by the x% of the volume; CI, conformity index; HI, homogeneity index; V_X_, volume receiving at least x Gy; LV, left ventricle; LAD, left anterior descending artery; MU, monitor unit; IMRT-ISO1, intensity-modulated radiation therapy with a single isocenter; IMRT-ISO2, intensity-modulated radiation therapy with two isocenters; VMAT-ISO1, volumetric-modulated arc therapy with a single isocenter; VMAT-ISO2, volumetric-modulated arc therapy with two isocenters. Bold represents p < 0.05*.

Based on these data, IMRT-ISO1 and VMAT-ISO2 plans were selected for further dosimetric comparisons with IMPT and HT.

### Dose Comparisons for PTVs

**Figure 1:**
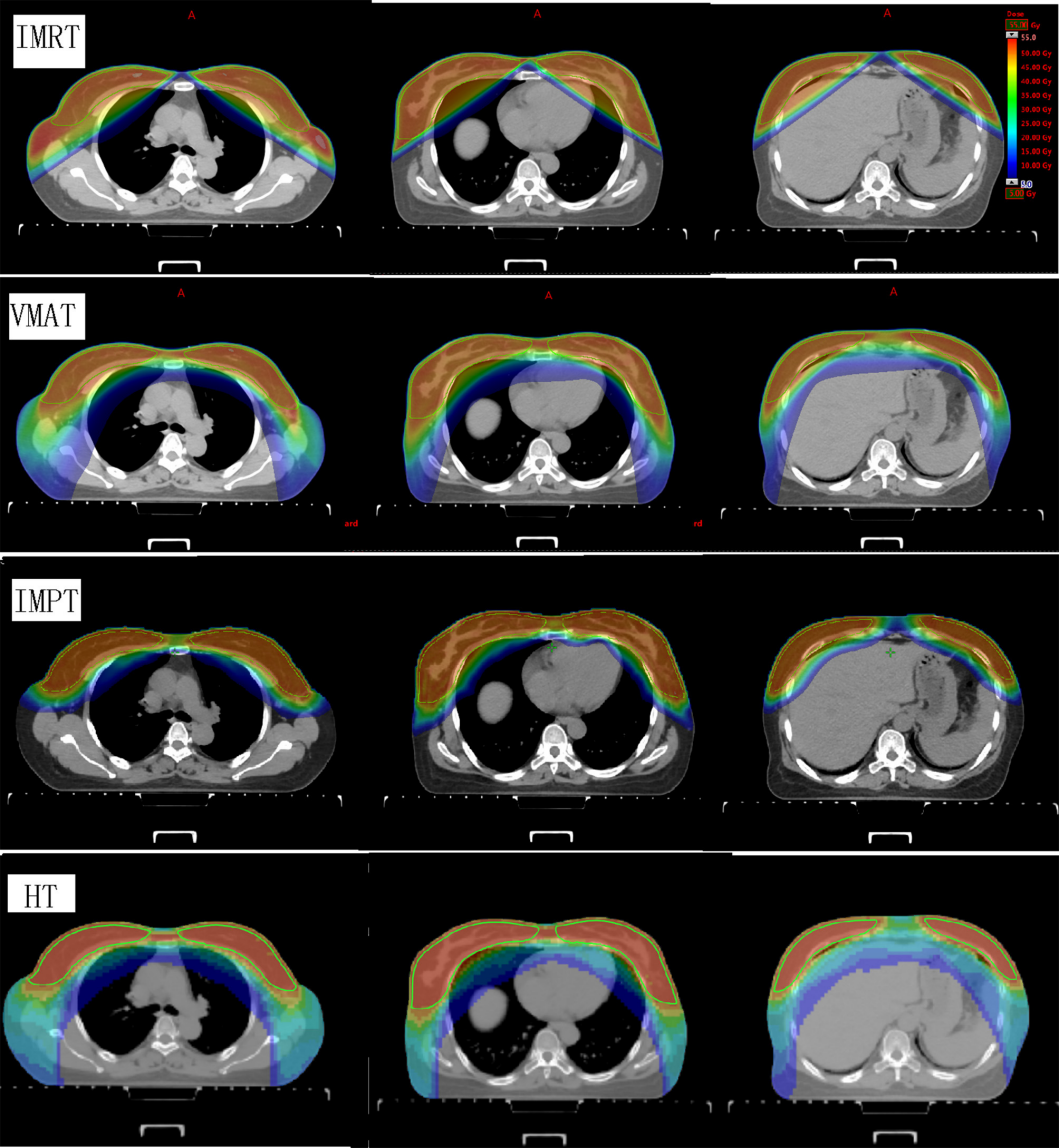
Dose distributions of the four plans for a representative patient. The images show IMRT, VMAT, IMPT, and HT techniques from the top row to the bottom row. The structures outlined in green are the bilateral whole breast planning target volumes. IMRT, intensity-modulated radiation therapy; VMAT, volumetric-modulated arc therapy; IMPT, intensity-modulated proton therapy; HT, helical tomotherapy.

**Figure 2:**
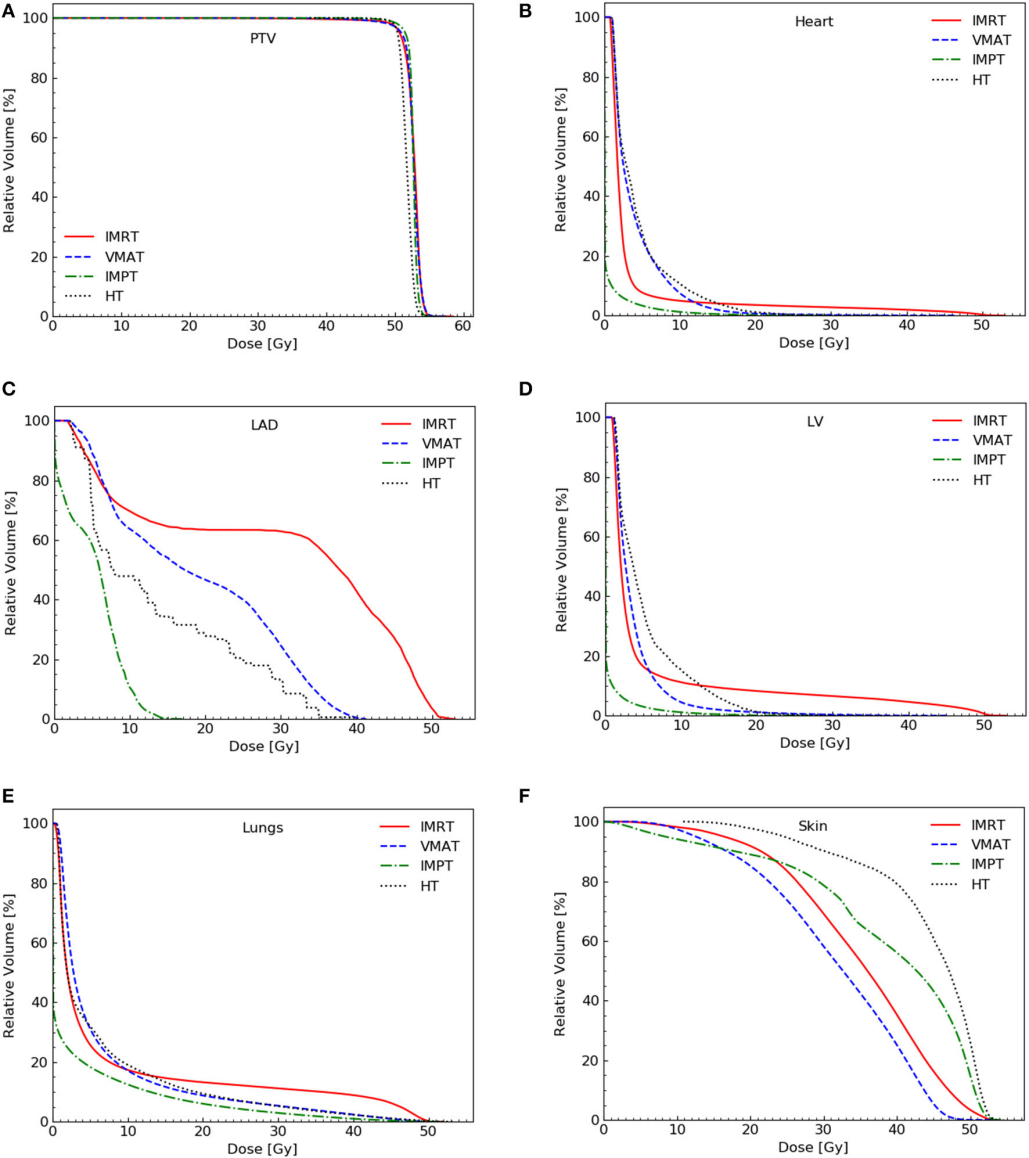
Dose-volume histograms for the four plans for a representative patient. PTV **(A)**, heart **(B)**, LAD **(C)**, LV **(D)**, lungs **(E)**, and skin **(F)**. PTV, planning target volume; LV, left ventricle; LAD, left anterior descending artery.

The dose coverage (V_100_) of the PTV was significantly better in IMPT plans than in all other plans (Table 2). No significant difference in the V_100_ was observed between the VMAT, IMRT, and HT plans. Among the four techniques, no significant differences were observed in the V_110_ of the PTV. IMPT and HT plans showed a lower maximum dose of the PTV (D_2_). VMAT plans showed the lowest minimum dose of the PTV (D_98_). VMAT and IMPT plans showed the best conformity, whereas IMRT plans showed the worst conformity. IMPT and HT plans achieved significantly higher dose homogeneity than IMRT and VMAT plans. Figure 1 shows the dose distributions of the four plans for a typical patient in our study. Figure 2 shows the DVHs of the four plans for the same patient.

**Table 2:** Summary of PTV dosimetric analysis.

	**IMRT**	**VMAT**	**IMPT**	**HT**	***p < 0.05***
V_100%_ (%)	96.9 ± 0.6	96.4 ± 0.8	97.6 ± 0.5	96.8 ± 0.4	b, d, f
V_110%_ (%)	0.23 ± 0.35	0.37 ± 0.39	0.17 ± 0.25	0.21 ± 0.18	–
D_2_ (cGy)	5,421.7 ± 32.3	5,430.9 ± 32.9	5,340.5 ± 52.0	5,330.7 ± 39.2	b, c, d, e
D_98_ (cGy)	4,918.7 ± 53.8	4,861.9 ± 78.3	4,963.9 ± 51.1	4,951.7 ± 38.9	d, e
CI	0.77 ± 0.04	0.84 ± 0.02	0.85 ± 0.04	0.80 ± 0.03	a, b, c, e, f
HI	0.10 ± 0.01	0.11 ± 0.02	0.08 ± 0.02	0.07 ± 0.01	b, c, d, e

*PTV, planning target volume; IMRT, intensity-modulated radiation therapy; VMAT, volumetric-modulated arc therapy; IMPT, intensity-modulated proton therapy; HT, helical tomotherapy; Vx_%_, volume receiving at least x% of the prescribed dose; Dx, dose received by the x% of the volume; CI, conformity index; HI, homogeneity index; a, IMRT vs. VMAT; b, IMRT vs. IMPT; c, IMRT vs. HT; d, VMAT vs. IMPT; e, VMAT vs. HT; f, IMPT vs. HT*.

### Dose Comparisons for the Heart and Substructures

Table 3 shows the dose parameters of the heart and substructures.

**Table 3:** Summary of the heart and substructure dosimetric analysis.

	**IMRT**	**VMAT**	**IMPT**	**HT**	***p < 0.05***
**Heart**					
V_5_ (%)	16.5 ± 10.4	29.5 ± 8.3	5.6 ± 2.0	29.6 ± 8.8	a, b, c, d, f
V_10_ (%)	9.2 ± 5.9	11.7 ± 6.1	2.9 ± 1.4	10.5 ± 3.6	b, d, f
V_20_ (%)	6.1 ± 3.6	2.4 ± 2.1	0.9 ± 0.7	2.0 ± 1.3	a, b, c, d, f
V_30_ (%)	3.9 ± 3.3	0.8 ± 1.3	0.8 ± 1.8	0.6 ± 0.9	a, b, c
V_40_ (%)	3.0 ± 2.6	0.1 ± 0.2	0.0 ± 0.1	0.2 ± 0.4	a, b, c
D_mean_ (cGy)	498.4 ± 184.7	484.7 ± 108.0	94.6 ± 31.4	482.9 ± 81.9	b, d, f
**LV**					
V_5_ (%)	26.8 ± 9.4	25.3 ± 13.7	9.1 ± 4.1	31.3 ± 11.8	b, d, f
V_10_ (%)	16.7 ± 6.7	8.3 ± 5.4	5.4 ± 3.1	12.9 ± 9.9	a, b, f
V_20_ (%)	12.3 ± 6.3	2.2 ± 2.0	1.7 ± 1.4	2.7 ± 1.6	a, b, c, f
V_30_ (%)	9.6 ± 6.1	0.8 ± 1.0	0.6 ± 0.6	0.6 ± 0.8	a, b, c
V_40_ (%)	6.8 ± 5.5	0.2 ± 0.3	0.1 ± 0.2	0.1 ± 0.2	a, b, c
D_mean_ (cGy)	778.1 ± 276.9	455.8 ± 129.8	154.7 ± 61.6	526.2 ± 103.9	a, b, c, d, e, f
**LAD**					
V_5_ (%)	76.9 ± 11.0	71.8 ± 23.1	41.3 ± 17.9	51.5 ± 20.6	b, c, d, e, f
V_10_ (%)	58.7 ± 21.1	53.0 ± 20.4	19.9 ± 9.0	34.4 ± 18.6	b, c, d, e, f
V_20_ (%)	47.3 ± 25.6	27.9 ± 19.8	3.2 ± 2.9	14.1 ± 10.4	a, b, c, d, e, f
V_30_ (%)	43.1 ± 26.3	13.5 ± 15.8	0.1 ± 0.3	5.3 ± 7.4	a, b, c, d, f
V_40_ (%)	21.3 ± 23.8	3.4 ± 10.6	0 ± 0	0.1 ± 0.4	a, b, c, d, e
D_mean_ (cGy)	2,149.7 ± 857	1,436.1 ± 576	537.2 ± 182.7	988.9 ± 344.8	a, b, c, d, e, f
D_max_ (cGy)	4,725.0 ± 504.6	3,886.8 ± 727.2	2,757.6 ± 509.7	3,433.9 ± 372.0	a, b, c, d, f

*IMRT, intensity-modulated radiation therapy; VMAT, volumetric-modulated arc therapy; IMPT, intensity-modulated proton therapy; HT, helical tomotherapy; V_X_, volume receiving at least x Gy; LV, left ventricle; LAD, left anterior descending artery; a, IMRT vs. VMAT; b, IMRT vs. IMPT; c, IMRT vs. HT; d, VMAT vs. IMPT; e, VMAT vs. HT; f, IMPT vs. HT*.

IMPT plans showed the lowest values for the V_5_, V_10_, V_20_, and D_mean_ of the heart (*p* < 0.05). The VMAT and HT plans showed the highest values for the V_5_ of the heart. IMRT significantly increased the V_20_, V_30_, and V_40_ of the heart compared with other techniques. No statistically significant differences were found in the V_10_ and D_mean_ of the heart among the VMAT, IMRT, or HT techniques. No statistically significant differences were found in the V_30_ and V_40_ of the heart among the VMAT, IMPT, or HT techniques.

Compared to other plans, IMPT plans reduced the V_5_ and mean dose of the LV significantly (*p* < 0.05). IMPT reduced the V_10_ and V_20_ of the LV slightly but non-significantly compared with VMAT (IMPT plans showed the lowest average values). IMRT plans showed the highest values for the V_20_, V_30_, V_40_, and D_mean_ of the LV. No statistically significant differences were found in the V_30_ and V_40_ of the LV among the VMAT, IMPT, or HT techniques.

A significant decrease in the V_5_, V_10_, V_20_, V_30_, D_mean_, and D_max_ of the LAD was achieved with the IMPT technique compared with the other techniques (*p* < 0.01). IMRT significantly increased the V_20_, V_30_, V_40_, D_mean_, and D_max_ of the LAD compared with the other techniques. The VMAT and IMRT plans showed the highest values for the V_5_ and V_10_ of the LAD. The V_40_ to the LAD was significantly lower in IMPT and HT than in the IMRT and VMAT techniques (*p* < 0.05).

### Dose Comparisons for OARs

For whole lungs, IMPT plans reduced the V_5_, V_10_, V_20_, and mean dose significantly compared with the other plans (*p* < 0.05). IMPT plans also showed the lowest average values for the V_30_ and V_40_ of the lungs among the four techniques, but no statistically significant differences were found compared with VMAT and HT. The VMAT and HT plans showed the highest values for the V_5_ of the lungs. IMRT plans increased the V_20_, V_30_, V_40_, and D_mean_ of the lungs significantly compared with other techniques. Table 4 shows the dose parameters of the lungs.

**Table 4:** Summary of lungs and liver dosimetric analysis.

	**IMRT**	**VMAT**	**IMPT**	**HT**	***p* < 0.05**
**Lungs**					
V_5_ (%)	30.2 ± 4.2	33.9 ± 3.4	19.8 ± 2.7	33.1 ± 3.6	a, b, c, d, f
V_10_ (%)	21.0 ± 3.6	20.5 ± 3.6	14.3 ± 2.4	19.9 ± 4.1	b, d, f
V_20_ (%)	15.6 ± 3.5	10.2 ± 2.5	7.9 ± 1.7	9.6 ± 2.6	a, b, c, d, f
V_30_ (%)	11.4 ± 4.1	6.0 ± 2.5	5.0 ± 3.3	6.0 ± 3.3	a, b, c, f
V_40_ (%)	9.0 ± 3.3	2.8 ± 1.3	1.8 ± 1.0	2.2 ± 1.2	a, b, c, d
D_mean_ (cGy)	881.8 ± 147.0	719.1 ± 89.2	414.8 ± 64.8	710.9 ± 98.9	a, b, c, d, f
**Liver**					
D_mean_ (cGy)	400.1 ± 140.0	363.8 ± 99.5	158.2 ± 92.8	483.9 ± 109.4	b, c, d, e, f

*IMRT, intensity-modulated radiation therapy; VMAT, volumetric-modulated arc therapy; IMPT, intensity-modulated proton therapy; HT, helical tomotherapy; V_X_, volume receiving at least x Gy; a, IMRT vs. VMAT; b, IMRT vs. IMPT; c, IMRT vs. HT; d, VMAT vs. IMPT; e, VMAT vs. HT; f, IMPT vs. HT*.

For the skin, statistically significant differences were found in the V_30_, V_40_, V_45_, and D_mean_ of the skin when comparing any two techniques. HT plans increased the V_30_, V_40_, V_45_, V_50_, and D_mean_ of the skin significantly compared with the other techniques. IMPT plans increased the V_30_, V_40_, V_45_, and D_mean_ of the skin significantly compared with IMRT and VMAT techniques. A significant decrease in the V_30_, V_40_, V_45_, V_50_, and D_mean_ of the skin was achieved with the VMAT technique compared with the other techniques (*p* < 0.01). Table 5 shows the dose parameters of the skin.

**Table 5:** Summary of skin dosimetric analysis.

	**IMRT**	**VMAT**	**IMPT**	**HT**	***p* < 0.05**
V_30_ (%)	75.2 ± 7.7	64.9 ± 8.8	81.8 ± 4.9	92.1 ± 2.4	a, b, c, d, e, f
V_40_ (%)	42.6 ± 10.1	29.8 ± 7.0	58.1 ± 6.6	85.2 ± 4.3	a, b, c, d, e, f
V_45_ (%)	22.9 ± 7.1	7.7 ± 2.6	42.2 ± 5.5	73.1 ± 7.6	a, b, c, d, e, f
V_50_ (%)	6.1 ± 3.1	0.1 ± 0.1	10.5 ± 3.7	28.7 ± 8.0	a, c, d, e, f
D_mean_ (cGy)	3,678.6 ± 226.3	3,317.9 ± 189.1	3,881.9 ± 172.3	4,287.6 ± 375.1	a, b, c, d, e, f

*IMRT, intensity-modulated radiation therapy; VMAT, volumetric-modulated arc therapy; IMPT, intensity-modulated proton therapy; HT, helical tomotherapy; V_X_, volume receiving at least x Gy; a, IMRT vs. VMAT; b, IMRT vs. IMPT; c, IMRT vs. HT; d, VMAT vs. IMPT; e, VMAT vs. HT; f, IMPT vs. HT*.

For the liver, IMPT plans reduced and HT plans increased the mean dose significantly compared with the other plans (*p* < 0.01). Table 4 shows the average mean dose of the liver in the four techniques.

Table 6 shows the dose parameters of B-P. For the B-P, IMPT plans reduced the dose (V_5_, V_10_, V_20_, V_30_, V_40_, V_50_, and D_mean_) significantly compared with the other plans. HT plans increased the V_5_, V_10_, V_20_, and D_mean_ of the B-P significantly, and IMRT plans increased the V_30_, V_40_, and V_50_ of the B-P significantly.

**Table 6:** Summary of B-P dosimetric analysis.

	**IMRT**	**VMAT**	**IMPT**	**HT**	***p* < 0.05**
V_5_ (%)	18.2 ± 2.9	26.0 ± 1.3	14.0 ± 1.5	28.9 ± 4.0	a, b, c, d, e, f
V_10_ (%)	14.4 ± 2.2	18.2 ± 2.3	11.3 ± 1.2	21.1 ± 2.8	a, b, c, d, e, f
V_20_ (%)	11.1 ± 1.7	11.3 ± 1.4	8.2 ± 0.9	12.9 ± 1.9	b, c, d, e, f
V_30_ (%)	8.8 ± 1.3	7.6 ± 0.9	6.0 ± 1.7	7.7 ± 1.0	a, b, c, d, f
V_40_ (%)	6.4 ± 1.0	4.5 ± 0.6	4.0 ± 0.5	4.5 ± 0.5	a, b, c, d, f
V_50_ (%)	2.0 ± 0.3	1.1 ± 0.2	1.2 ± 0.3	1.3 ± 0.2	a, b, c, e, f
D_mean_ (cGy)	604.0 ± 78.3	659.1 ± 67.5	394.6 ± 43.0	704.8 ± 78.0	a, b, c, d, e, f

*B-P, body minus the PTV; IMRT, intensity-modulated radiation therapy; VMAT, volumetric-modulated arc therapy; IMPT, intensity-modulated proton therapy; HT, helical tomotherapy; V_X_, volume receiving at least x Gy; a, IMRT vs. VMAT; b, IMRT vs. IMPT; c, IMRT vs. HT; d, VMAT vs. IMPT; e, VMAT vs. HT; f, IMPT vs. HT*.

## Discussion

SBBC is a rare disease. There are no standard guidelines for the treatment of SBBC, and due to the increased demand for breast-conserving treatment, synchronous irradiation of the bilateral breast is imperative. Synchronous bilateral breast irradiation is challenging due to the large and complex target volume and the need to minimize the dose to the heart and lungs. IMRT, VMAT, and HT techniques have been reported to improve dosimetry in SBBC patients, and past dosimetric studies have shown that IMPT can reduce the cardiac dose significantly in unilateral breast cancer (11–19). Previous studies have not compared dosimetric differences between IMRT, VMAT, and HT in SBBC. To the best of our knowledge, this is the first report on the dosimetry of IMRT, VMAT, HT, and IMPT for SBBC.

First, we compared the dosimetric differences between single- and dual-isocentric IMRT and VMAT plans. Compared to the IMRT-ISO2 plans, significantly higher dose homogeneity and heart dose sparing were achieved with IMRT-ISO1 plans. VMAT-ISO2 plans showed higher conformity and significantly reduced lung, heart, LV, and LAD doses than VMAT-ISO1 plans. Boman et al. (27) compared mono-isocenter and dual-isocenter VMAT plans for 11 SBBC patients with lymph node positivity. When compared to Iso1 plans, Iso2 plans reduced the mean dose for the lungs and heart from 11.3 Gy and 3.8 Gy to 10.9 Gy and 3.6 Gy, respectively. In single-isocenter plans, there was a statistically positive correlation between the PTV volume and the maximum PTV dose, but this was not observed in the dual-isocenter plans. This suggests that the use of a single isocenter may not be the best solution for very large bilateral breasts and that a dual-isocentric solution may be required. However, the LAD and LV were not delineated in the above study. In our study, paring apple-like tangential beams were designed for VMAT plans to minimize the exposure of the lungs and heart. The medial x-jaw shielding of the lungs and heart was set to the minimum site (−2 cm). Because the minimum setting of the medial x-jaw value can only be −2 cm, VMAT-ISO2 plans were better at blocking the lungs and heart than VMAT-ISO1 plans. Therefore, the VMAT-ISO2 plans in this study had better dosimetric advantages. The IMRT plans were mainly based on tangential fields, which is similar to the field-in-field technique. Therefore, the IMRT-ISO2 plans did not show a dosimetric advantage, and IMRT-ISO1 plans were superior. IMRT-ISO1 and VMAT-ISO2 plans were selected for further dosimetric comparisons with IMPT and HT plans.

IMPT plans achieved the highest dose coverage of the PTV compared with all other plans. VMAT and IMPT plans showed the best conformity, whereas IMRT plans showed the worst conformity. IMPT and HT plans achieved significantly higher dose homogeneity and a lower maximum dose of the PTV (D_2_) than IMRT and VMAT plans. The dosimetric advantage for the target of proton therapy has been recognized (16–19). In our study, we found that VMAT, IMRT, and HT techniques achieved comparable dose coverage of the PTV.

Despite significant reductions in cardiac doses over the past few years (6, 28), radiation-induced heart disease remains the leading cause of death among long-term breast cancer survivors after radiation therapy. Darby et al. (2) found that there was a linear relationship between the MHD and the incidence of ischemic heart disease, which increased by 7.4% per Gy of the MHD. Therefore, the MHD is commonly used as a reference for cardiotoxicity studies. However, there is increasing evidence that the dose of heart substructures needs to be considered. Some studies have focused on the LAD and LV as important parts of the heart associated with radiation-induced heart disease (8, 29). Therefore, an appropriate technique that could minimize cardiac and substructure doses in breast cancer radiation therapy may be beneficial for breast patients. Proton therapy has been confirmed to reduce the dose of the heart and LAD in patients with left breast cancer especially because high cardiac doses remain in advanced photon techniques (17–19). In our study, we also found a dosimetric advantage of IMPT for sparing the heart, LAD, and LV doses in SBBC patients. IMPT plans reduced the mean dose and low dose volume of the heart, LAD, and LV. In high dose volumes of the heart and cardiac substructures, IMPT, VMAT, and HT techniques showed similar advantages, and IMRT plans increased the values more than other techniques. IMPT is the only technique capable of delivering an MHD <1 Gy, whereas the three other techniques delivered <5 Gy. Kim et al. (15) compared 3DCRT, IMRT, and VMAT treatment plans for 10 SBBC patients. In their study, 3DCRT reduced the mean dose and V_10Gy_ of the heart, and IMRT reduced the V_20Gy_ and V_40Gy_ of the heart. These results are different from the results of our study mainly because of the different irradiation field angles. In their study, 3DCRT comprised eight fields with multiple isocenters, whereas IMRT and VMAT comprised 12 fields and two partial arcs with a single isocenter. The increase in the number and angle of irradiation fields will increase the exposure to a low dose and thus increase the cardiac mean dose. In their study, the D_mean_ of the heart in 3DCRT, IMRT, and VMAT were 818.00 ± 306.09 cGy, 946.08 ± 166.75 cGy, and 1,447.65 ± 239.94 cGy, respectively. The values in our study were lower than the values in their study probably because of the fields that we used. In our study, no breath-holding was used. To further reduce the dose to the heart, deep-inspiration breath hold (DIBH) could be used in IMRT and VMAT plans.

Past studies have demonstrated that proton therapy can significantly reduce the V_5_ and V_20_ values in the ipsilateral lung by nearly 50% compared to traditional 3D-CRT and IMRT, and the low-dose radiation volume could be reduced significantly while providing a reduced or similarly high-dose radiation volume with proton therapy in unilateral breast cancer (19, 30, 31). In our study, IMPT plans reduced the mean dose and all dose-volume parameters of lungs. Lung cancer data showed a correlation between the multi-dose-volume parameters and late pulmonary fibrosis. The dosimetric factors of the mean lung dose and the V_10Gy_, V_20Gy_, V_30Gy_, V_40Gy_, and V_50Gy_ of the lung were highly correlated with late radiation fibrosis (32). Therefore, IMPT may reduce the incidence of late pulmonary complications compared to the photon techniques. In the four techniques, the values of the V_20_ for the lungs did not exceed 20%. The VMAT and HT plans increased the low dose (V_5_), and the IMRT plans increased the high dose (V_20_, V_30_, and V_40_) and D_mean_ of the lungs significantly. No differences were observed between VMAT and HT techniques in all dose-volume parameters of the lungs.

Compared with photon therapy, the disadvantages of relatively high skin doses and different levels of dermatitis have been reported in proton therapy clinical studies (33, 34). Therefore, in this study, the skin dose was included in the results. The results showed that IMPT plans increased the skin dose significantly compared with IMRT and VMAT techniques. However, HT plans showed the highest skin dose in the four techniques, and the lowest dose was achieved in VMAT plans. In our study, we did not optimize the skin dose. Tommasino et al. (35) published a model-based approach that showed that optimizing skin doses in IMPT may reduce the risk of acute skin toxicity compared to IMRT. If we add the skin to the optimization process, the skin dose could be reduced in the four techniques. This will be examined in future research.

IMPT plans reduced the mean dose of the liver significantly, and HT plans increased the mean dose compared with other plans (*p* < 0.01). The HT plans showed the highest liver dose, but the D_mean_ was <5 Gy. Patients with radiation-induced liver disease (RILD) receive a significantly higher mean dose to the liver, and the incidence rate of RILD was <5% when the dose of the whole liver was <30–32 Gy (36). So, we believe that in terms of liver protection, all four techniques are feasible.

IMPT plans reduced the dose of non-target tissues significantly compared with other plans. The reduction in these doses may result in a decreased incidence of secondary malignancies after adjuvant radiotherapy (37).

As discussed above, IMPT plans achieved superior cardiac and lung sparing in synchronous bilateral breast radiotherapy compared to photon therapy, which may lead to a reduction in cardiopulmonary toxicity and the potential benefits related to this reduction. Because proton therapy is still a less common and more expensive treatment technique, VMAT and HT could be used as a suboptimal technique for SBBC patients. DIBH could be used in photon plans to reduce the dose to the heart and lungs.

## Conclusion

In SBBC radiotherapy, IMPT plans improve both the target coverage and OARs sparing, especially for the heart, cardiac substructures (LAD and LV), lungs, and normal tissue. Long-term follow-up is required to confirm the benefits of IMPT over photon techniques for late toxicity and secondary malignancies. IMRT technique increases the mean dose and high dose volume of all OARs while only reducing the V_5_ of the lungs and heart compared to VMAT and HT techniques. VMAT and HT techniques show better dosimetric advantages compared to IMRT technique. VMAT and HT could be used as a suboptimal technique for SBBC patients.

## Data Availability Statement

The datasets generated for this study are available on request to the corresponding author.

## Ethics Statement

This study was approved by the Research Ethics Board of the Shandong Cancer Hospital. Written informed consent was not required in accordance with national and institutional guidelines.

## Author Contributions

TS and YY designed the study. YT collected the CT data and delineated OARs volumes. TS, XLin, and XLiu designed the treatment plans. LP, CT, and JD analyzed the data. TS wrote the paper. All authors read and approved the final manuscript.

### Conflict of Interest

The authors declare that the research was conducted in the absence of any commercial or financial relationships that could be construed as a potential conflict of interest.
